# Functional regeneration of the murine neuromuscular synapse relies on long-lasting morphological adaptations

**DOI:** 10.1186/s12915-022-01358-4

**Published:** 2022-07-08

**Authors:** Francisca Bermedo-García, Diego Zelada, Esperanza Martínez, Lucía Tabares, Juan Pablo Henríquez

**Affiliations:** 1 Laboratory of Neuromuscular Studies (NeSt Lab), Group for the Study of Developmental Processes (GDeP), Department of Cell Biology, Faculty of Biological Sciences, Universidad de Concepción, Concepción, Chile; 2 Department of Medical Physiology and Biophysics, School of Medicine, Universidad de Sevilla, Sevilla, Spain

**Keywords:** Neuromuscular junction, Presynaptic, Postsynaptic, Denervation, Regeneration, Fragmentation, Terminal Schwann Cells, Poly-innervation

## Abstract

**Background:**

In a broad variety of species, muscle contraction is controlled at the neuromuscular junction (NMJ), the peripheral synapse composed of a motor nerve terminal, a muscle specialization, and non-myelinating terminal Schwann cells. While peripheral nerve damage leads to successful NMJ reinnervation in animal models, muscle fiber reinnervation in human patients is largely inefficient. Interestingly, some hallmarks of NMJ denervation and early reinnervation in murine species, such as fragmentation and poly-innervation, are also phenotypes of aged NMJs or even of unaltered conditions in other species, including humans. We have reasoned that rather than features of NMJ decline, such cellular responses could represent synaptic adaptations to accomplish proper functional recovery. Here, we have experimentally tackled this idea through a detailed comparative study of the short- and long-term consequences of irreversible (chronic) and reversible (partial) NMJ denervation in the convenient cranial *levator auris longus* muscle.

**Results:**

Our findings reveal that irreversible muscle denervation results in highly fragmented postsynaptic domains and marked ectopic acetylcholine receptor clustering along with significant terminal Schwann cells sprouting and progressive detachment from the NMJ. Remarkably, even though reversible nerve damage led to complete reinnervation after 11 days, we found that more than 30% of NMJs are poly-innervated and around 65% of postsynaptic domains are fragmented even 3 months after injury, whereas synaptic transmission is fully recovered two months after nerve injury. While postsynaptic stability was irreversibly decreased after chronic denervation, this parameter was only transiently affected by partial NMJ denervation. In addition, we found that a combination of morphometric analyses and postsynaptic stability determinations allows discriminating two distinct forms of NMJ fragmentation, stable-smooth and unstable-blurred, which correlate with their regeneration potential.

**Conclusions:**

Together, our data unveil that reversible nerve damage imprints a long-lasting reminiscence in the NMJ that results in the rearrangement of its cellular components. Instead of being predictive of NMJ decline, these traits may represent an efficient adaptive response for proper functional recovery. As such, these features are relevant targets to be considered in strategies aimed to restore motor function in detrimental conditions for peripheral innervation.

**Supplementary Information:**

The online version contains supplementary material available at 10.1186/s12915-022-01358-4.

## Background

The neuromuscular junction (NMJ) is the peripheral synapse responsible for skeletal muscle contraction. The mature vertebrate NMJ is composed of a motor nerve terminal, a specialized acetylcholine receptor (AChR)-enriched fraction of the postsynaptic muscle membrane, and non-myelinating terminal Schwann cells (tSCs) [1]. The NMJ is subjected to denervation due to traumatic injuries to peripheral nerves or pathologies. Even though the peripheral nervous system displays a comparatively higher regenerative capacity than the central nervous system, this capability is strongly dependent on the establishment of permissive cellular and molecular niches at the NMJ and the denervation-reinnervation time frame [2]. Therefore, a deep understanding of the cellular responses that allow proper synaptic function recovery is critical to design strategies to repair and/or to prevent the degeneration of the neuromuscular synapse during the denervation timespan. Experimental paradigms of peripheral nerve injury have shown nerve—muscle connectivity restoration after partial nerve damage [3, 4]. In this regard, the sequence of the key cellular processes leading to successful NMJ reinnervation has been well described, particularly in limb muscles of murine species [5]. After initial denervation of motor endplates due to Wallerian degeneration of the distal axon stump, mediated by trans-differentiated Schwann cells (SCs) and macrophages, SCs rearrange into Büngner bands that guide regenerating motor axons towards denervated muscles around 2 weeks after injury [6]. Subsequently, nascent poly-innervated NMJs turn into functional mono-innervated NMJs 6 weeks after nerve injury [5]. The fine-tuning of NMJ reinnervation relies on the ability of tSCs to extend transient cell processes that guide the regenerated motor axons to the denervated postsynaptic domains with high precision to avoid axonal miswiring [7, 8]. Indeed, cranial nerves misguiding results in synkinesis of facial muscles [9]. In the postsynaptic domain, despite the gross morphological maintenance of AChR distribution after nerve injury, their stability in the muscle membrane decreases soon after denervation [10] and postsynaptic fragmentation is observed after nerve cut followed by repair [11].

Even though the cellular events occurring during reversible NMJ denervation configure a fingerprint of its early regeneration, some of these phenotypes are also observed in mice models of muscular and neurological pathologies. These include NMJ postsynaptic fragmentation [12], tSC sprouting and migration outside the NMJ [13], and poly-innervation of the postsynaptic domains [14]. In addition, defining the hallmarks of NMJ regeneration becomes more complex considering that some of its cellular features can also be observed at the NMJ in physiological conditions. For instance, a significant proportion of healthy NMJs (~ 20%) exhibit tSC sprouting [15], whereas fragmentation and poly-innervation of postsynaptic domains become increasingly prevalent in the NMJs of aged animals [1, 16, 17]. In murine species, while postsynaptic domains are poly-innervated at birth, they become mono-innervated during early postnatal development following a synaptic elimination process that occurs at different rates across muscles [18, 19]. Moreover, the NMJ of a variety of species, such as snakes, frogs, sheep, and humans, are significantly more fragmented and smaller than murine ones [20, 21]. Together, these findings suggest that specific morphological alterations in the three cellular components of the NMJ could be coordinated to exert an adaptive response to accomplish a proper synaptic function.

Considering that most studies have analyzed muscle function and morphology until NMJ reinnervation is achieved [5, 22], we reasoned that potential adaptive responses of the NMJ could be better visualized long after reinnervation occurs. Therefore, we have conducted a detailed characterization of the long-term consequences of NMJ short-term reinnervation (i.e., after partial denervation) and compared it to those occurring after irreversible (chronic) denervation. We have employed the cranial *levator auris longus* (LAL) muscle due to its high accessibility for morphometric and functional analyses [23], its suitability for short-term reinnervation [23], and its potential clinical relevance regarding the poor recovery outcomes observed in head muscles [24]. Our findings show that fragmentation and poly-innervation of the NMJ postsynaptic domain remain long after re-innervation and synaptic transmission recovered. Based on our analysis of NMJ morphology and postsynaptic stability, we also identified two fragmentation phenotypes associated with their regenerative potential. These results suggest that long-lasting morphological adaptations of the neuromuscular synapse allow functional recovery after nerve damage.

## Results

### Time course of changes in the three cellular components of the NMJ after chronic denervation

**Fig. 1:**
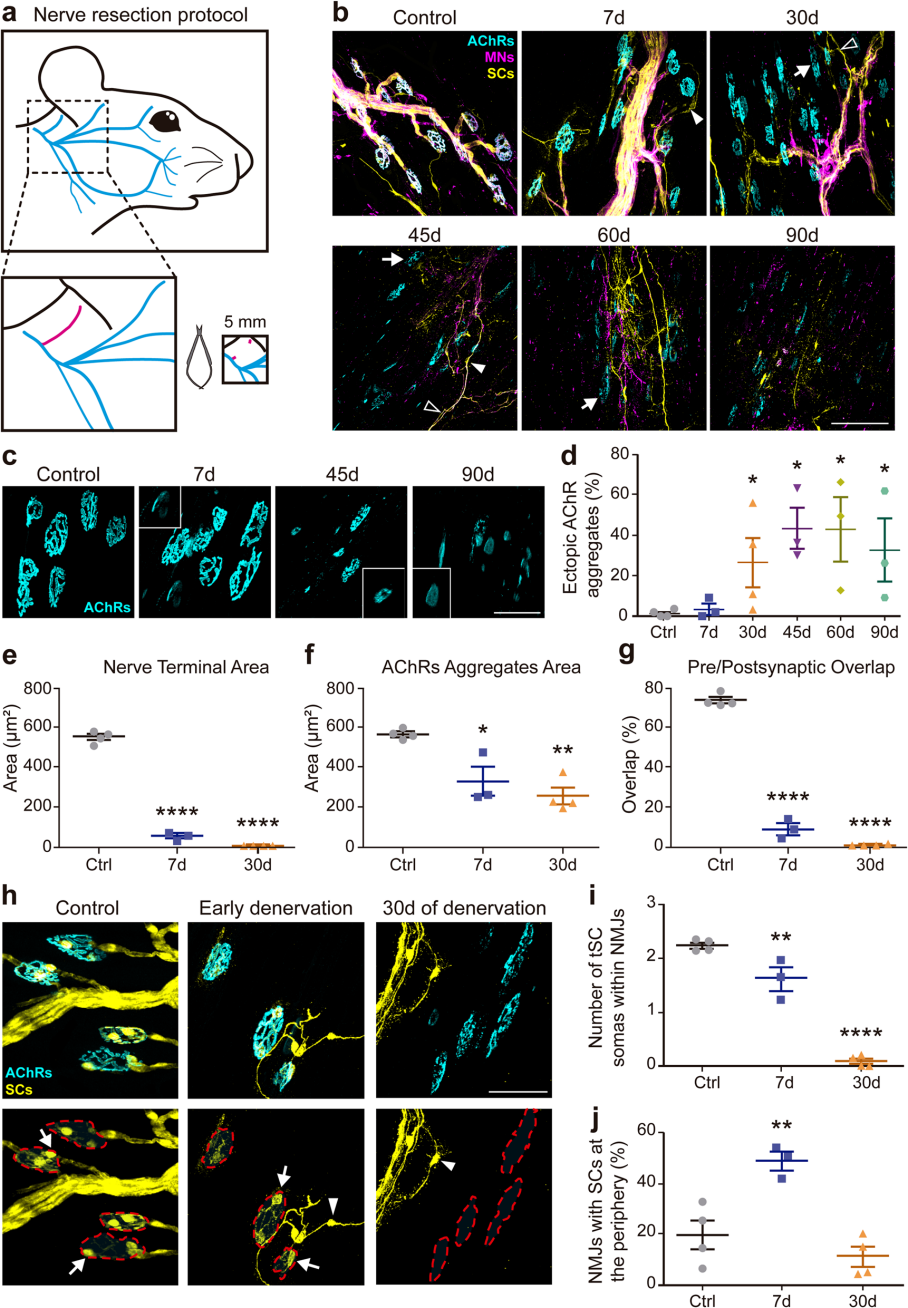
Alterations in NMJ morphology after distal chronic denervation of the LAL muscle. **a** Experimental design for the degenerative model. Approximately 5 mm of the posterior auricular branch of the facial nerve were resected. **b** Whole mount LAL muscles from control adult mice and mice 7, 30, 45, 60, and 90 days after nerve damage were dissected and processed for immunofluorescence staining with 2H3 (neurofilaments) plus SV2 (synaptic vesicles) antibodies to reveal motor axons and presynaptic terminals (magenta), with an anti S100B antibody to stain Schwann cells (yellow), and with BTX (cyan) to stain AChRs located at postsynaptic densities. Fragmented postsynaptic domains (arrows), detached tSCs (arrowheads), and tSC projections (empty arrowheads) are indicated. Bar = 100 μm. **c** Representative images of ectopic AChR aggregates at the indicated times after chronic NMJ denervation, AChR were labeled with BTX. Insets show higher magnification of ectopic AChR aggregates. Bar = 50 μm. **d** Quantification of the percentage of ectopic AChR aggregates at different times after denervation. **e**, **f** Nerve terminal and AChR aggregates area were measured at 7 and 30 days after nerve injury and in control muscles. **g** The overlap between pre and postsynaptic apparatuses was calculated based on nerve terminal and AChR areas. **h** tSCs behavior in NMJs of control (left panel), early denervated (middle panel), and 30 days of denervation (right panel) muscles. Lower panels show only the SC channel while the postsynaptic domain is delimited (dashed red line). Representative images show tSCs covering the NMJ (arrows) and tSC/SC located outside the NMJ (arrowheads). Bar = 50 μm. **i**, **j** Quantification of tSC per NMJ and tSC at the periphery (i.e., within a 50 μm perimeter from the postsynaptic region) of each NMJ. The results are represented as the mean ± SEM and each individual value (N: 3–4 mice; 2 female plus 1–2 male mice). *p < 0.05, Mann–Whitney test (**d**), **p < 0.01, ****p< 0.0001, one-way ANOVA test (**e**–**g**, **i**, **j**)

We analyzed the long-term morphological alterations associated with irreversible denervation of the cranial LAL muscle. Besides its usefulness to study the specific clinical consequences associated with the denervation of head/neck muscles [24], the LAL muscle offers several experimental advantages, as it is a superficially exposed, flat, and thin cranial muscle, allowing repeated in vivo manipulation and microscopic observation of NMJs in whole-mount preparations [23, 25, 26]. We have recently refined a muscle denervation procedure to specifically target muscles from the cranial region, by resecting a 5-mm segment of the right posterior auricular nerve branch (Fig. 1a), while isolateral muscles from non-injured animals were used as control [23]. Denervated LAL muscles were dissected at different times after nerve resection and stained to reveal the three cellular components of the NMJ. Control NMJs exhibited full innervation of pretzel-like AChR aggregates and were completely covered by tSCs (Fig. 1b). In turn, at different times after nerve resection, motor axons degenerate (evidenced by decreased staining in motor terminals and motor axons), postsynaptic domains went through morphological modifications from a pretzel-like to a fragmented shape (Fig. 1b, arrows), and tSCs migrated towards the vicinity of denervated NMJs (Fig. 1b, arrowheads) and projected elongated cellular processes (Fig. 1b, empty arrowheads).

We first studied the formation of ectopic AChR clusters, a postsynaptic hallmark of NMJ denervation [27]. As expected, we found that the percentage of ectopic AChR aggregates increased since 30 days after denervation, representing around 40% of total AChR aggregates, significantly higher than 1.42 ± 0.87 % obtained in controls (Fig. 1c, d). The increasingly high proportion of ectopic AChR aggregates correlated with drastic alterations in the gross morphology of the postsynaptic domains (Fig. 1c), making it unfeasible to recognize previously innervated NMJs. Therefore, the next analyses in the chronic denervation model were performed at 7 and 30 days after nerve resection. Both time points were characterized by a marked decrease in the measurement of presynaptic area, which likely corresponded to small remnants of axon terminal debris (Fig. 1e) and subsequent endplate denervation. The postsynaptic domain was also subjected to morphological changes, as the quantification of the area of AChR aggregates (excluding ectopic aggregates) showed a progressive decrease, already detectable 7 days after damage (Fig. 1f). Consequently, the apposition between pre and postsynaptic domains showed a nearly null overlap after nerve damage at both time points (7 and 30 days) (Fig. 1g).

We next analyzed the behavior of tSCs after NMJ chronic denervation. tSCs were identified by their positive S100B staining, their distribution on or in the vicinity of NMJs, and by their characteristic shape [7, 28, 29]. Indeed, soon after facial nerve resection (7 days), tSCs projected cell processes and their somas migrate out from the synaptic region (Fig. 1h, arrowheads), which led to a significant decrease in the average number of tSC somas at the NMJ (1.60 ± 0.38) compared to controls (2.30 ± 0.15; **p < 0.01, one-way ANOVA) (Fig. 1i). At longer denervation times (30 days), the average number of tSCs was less than 1 per NMJ (Fig. 1i); consequently, the number of tSCs located within a 50 μm radius of NMJs showed a significant increase shortly after denervation (7 days); however, the number of tSC in the periphery of the NMJ exhibits a trend to decrease 30 days after injury (Fig. 1j).

Collectively, our findings reveal that the cranial LAL muscle exhibits morphological responses in the three cellular components of the neuromuscular synapse after chronic denervation, comparable to those described in hind-limb muscles.

### Transient NMJ denervation alters the behavior of its cellular components

**Fig. 2:**
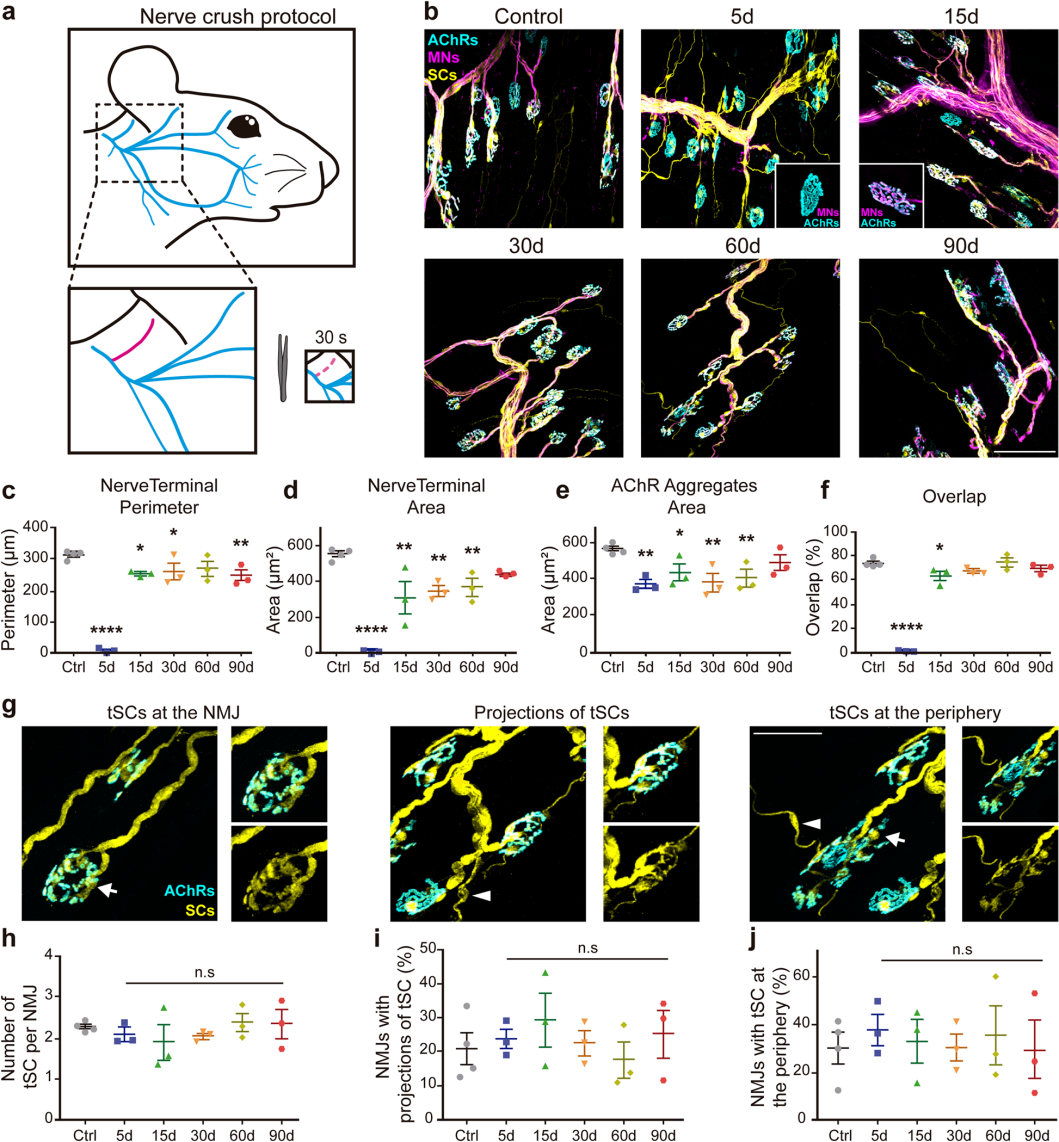
Morphological NMJ regeneration of the LAL muscle following distal nerve crush injury. **a** Experimental design for the regenerative model. The posterior auricular branch of the facial nerve was crushed for 30 seconds. **b** LAL muscles from control adult mice and mice 5, 15, 30, 60, and 90 days after nerve damage were dissected and processed for immunofluorescence staining with 2H3 (neurofilaments) plus SV2 (synaptic vesicles) antibodies to reveal motor axons and presynaptic terminals (magenta), with an anti S100B antibody to stain Schwann cells (yellow), and with BTX (cyan) to stain AChRs located at postsynaptic densities. Insets in 5d and 15d show only pre- and postsynaptic staining of NMJs. Bar = 100 μm. **c**–**f** Nerve terminal perimeter, nerve terminal area, AChR aggregates area, and overlap between pre and postsynaptic apparatuses were measured at different times after nerve crush injury and in control muscles. **g** The behavior of tSCs was analyzed by following tSCs covering the NMJ (arrows) (left panel), tSCs covering the NMJ and extending cell projections (middle panel), and tSC at the periphery (i.e., within a 50 μm perimeter from the postsynaptic region) of each NMJ (arrowheads) (right panel). Bar = 50 μm. **h**–**j** Quantification of tSC behavior after nerve crush injury. The results are represented as the mean ± SEM and each individual value (N: 3–4 mice; 2 female plus 1–2 male mice). *p < 0.05, **p < 0.01, ****p< 0.0001, one-way ANOVA test (**c**, **d**, **e**, **f**). n.s. = non-significant (**h**, **i**, **j**)

To study the behavior of the three NMJ cellular components upon reversible axonal damage, the right posterior auricular branch of the facial nerve was crushed for 30 s (Fig. 2a). Five days after nerve crush, most endplates were denervated (Fig. 2b, inset). Endplate re-innervation was detected 15 days after injury where reinnervated NMJs exhibited a similar shape that control muscles (Fig. 2b, inset). Quantification of presynaptic parameters showed that both nerve terminal perimeter and area were decreased to almost undetectable levels at the time previously described for Wallerian degeneration (5 days post-injury) (Fig. 2c, d) [5]. Interestingly, even though morphological parameters of the nerve terminal, such as perimeter (Fig. 2c) and area (Fig. 2d) recovered values since 30 days after injury, they remained significantly lower than control after reinnervation. As comparable long-lasting alterations were observed in the area of postsynaptic AChR aggregates (Fig. 2e), the simultaneous decrease in the size of both pre and postsynaptic domains gave rise to smaller NMJs than controls, allowing full NMJ reinnervation 30 days after nerve injury (Fig. 2f). Finally, to analyze if tSC behavior was altered as a consequence of short-term NMJ reinnervation, we quantified the number of tSCs per NMJ (Fig. 2g, h), the proportion of NMJs bearing tSC projections (Fig. 2g, i), and the proportion of NMJs having tSCs in the periphery (Fig. 2g, j). No significant alterations were found at the different times analyzed after reversible nerve injury compared to controls.

**Fig. 3:**
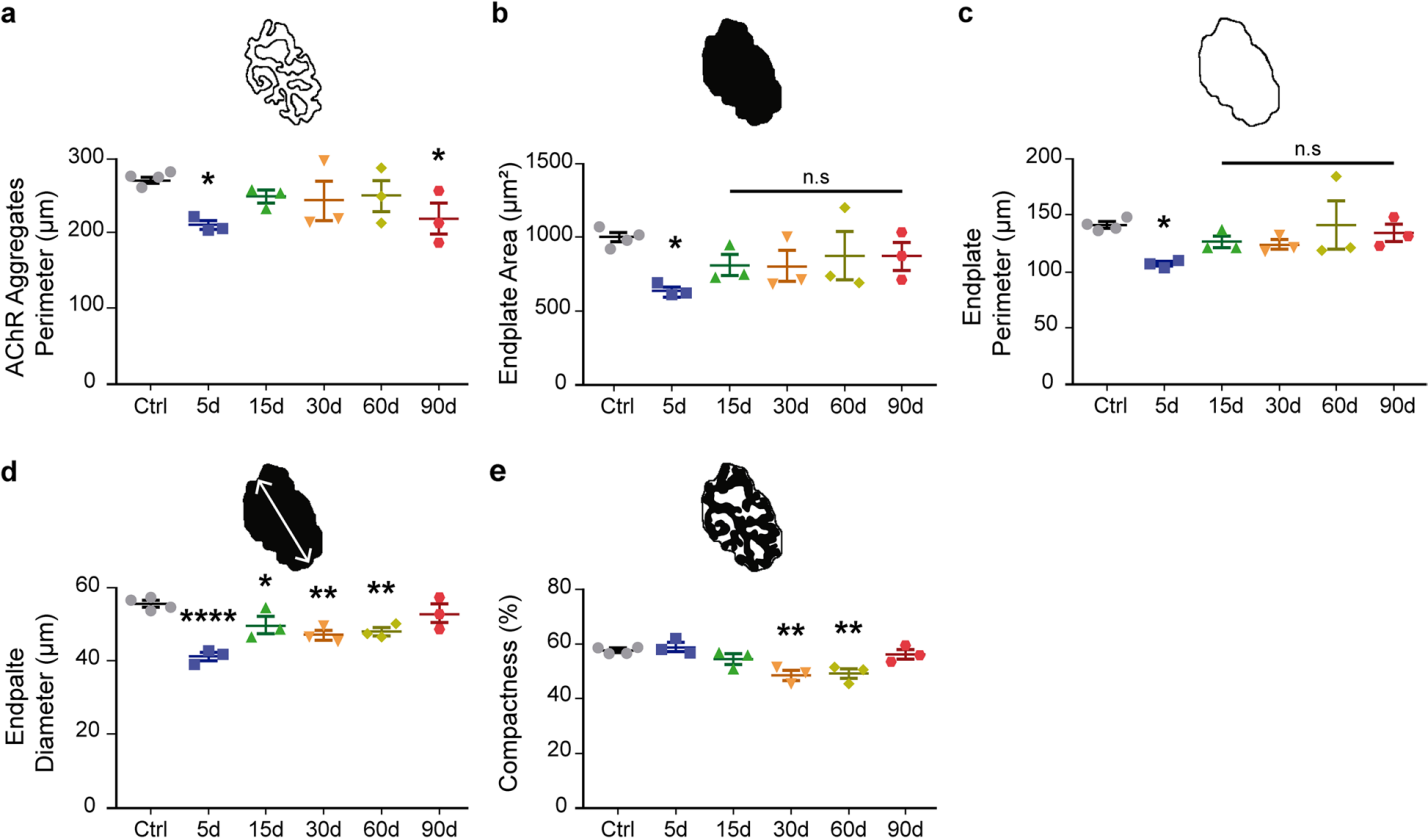
Morphological recovery of the NMJ postsynaptic domain during regeneration. Morphological parameters of the NMJ postsynaptic domain of LAL muscles were quantified 5, 15, 30, 60, and 90 days after nerve crush injury and in control mice. **a**–**e** AChR aggregates perimeter, endplate area, endplate perimeter, endplate diameter, and compactness of the postsynaptic domain. Drawings on top of each plot represent each of the measured parameters. The results are represented as the mean ± SEM and each individual value (N: 3–4 mice; 2 female plus 1–2 male mice). *p < 0.05, **p < 0.01, ****p< 0.0001, one-way ANOVA test. n.s. = non-significant

Based on previous findings showing that the postsynaptic organization is not significantly affected upon muscle denervation [5, 30], we next conducted detailed analyzes of the postsynaptic morphology at different times after reinnervation. The perimeter of AChR aggregates decreased immediately after nerve injury and recovered values similar to control after 2 weeks; however, a decrease was observed 3 months after injury (Fig. 3a). The endplate area decreased 5 days after damage to reach control values as soon as re-innervation was accomplished; similarly, the endplate perimeter transiently decreased but exhibited control values since 15 days post nerve injury (Fig. 3b, c). Other postsynaptic parameters, such as endplate diameter (Fig. 3d) and postsynaptic compactness (Fig. 3e), defined as the endplate area occupied by AChRs, were transiently and slightly decreased but exhibited control values 90 days post nerve injury. In sharp contrast with the degenerative model (Fig. 1d), even though few scatter ectopic AChR clusters were observed during early denervation, they were no longer detectable upon NMJ reinnervation, as described [27]. Altogether, our findings thus far reveal that some changes occurring upon NMJ denervation in the LAL muscle are rescued with short-term reinnervation, while others remain altered long after reinnervation.

### The stability of the NMJ postsynaptic domain is negatively affected by nerve damage

**Fig. 4:**
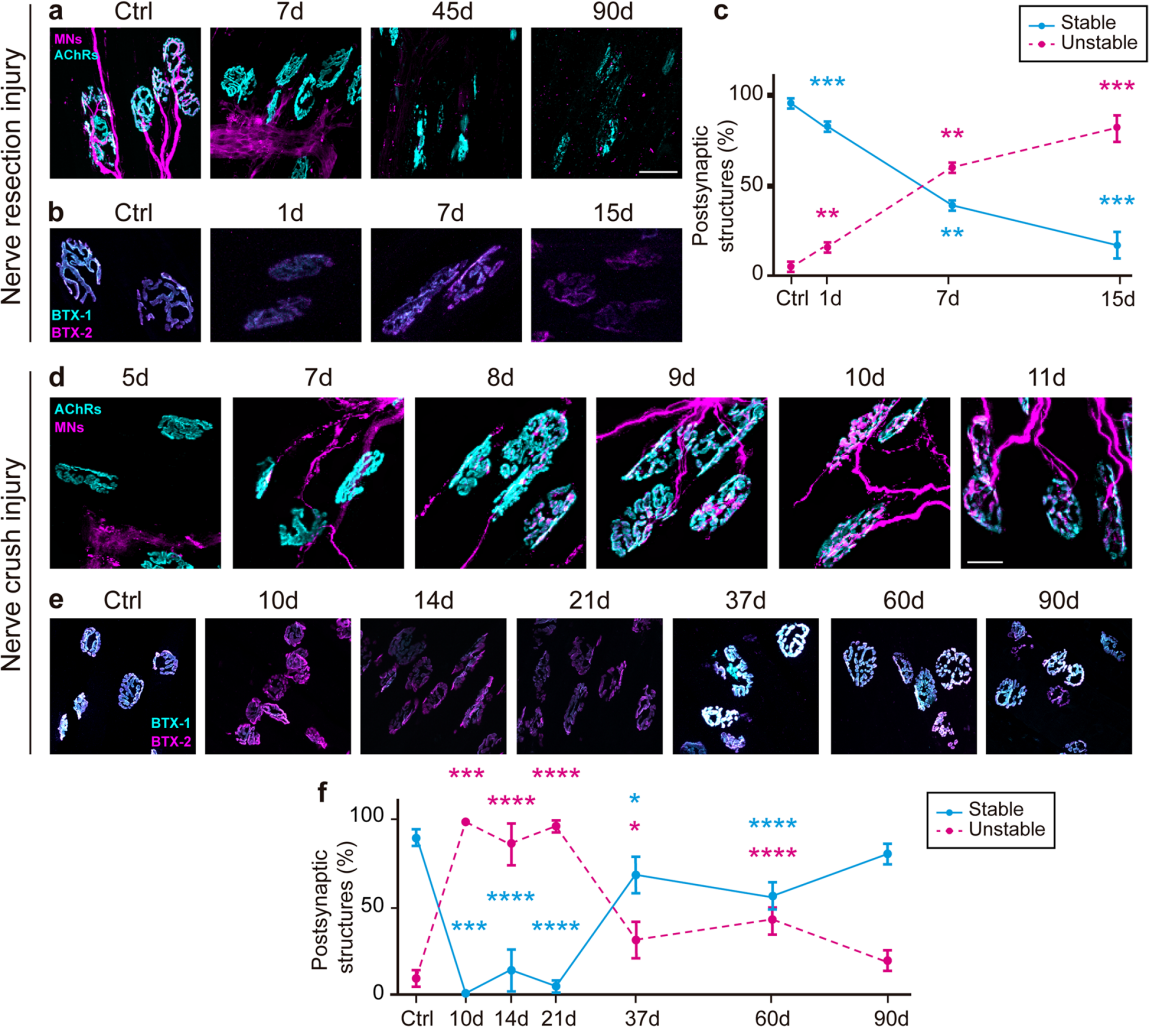
Stability of the NMJ postsynaptic domain after nerve injury. **a** LAL muscles were dissected from control mice and 7, 45, and 90 days after facial nerve resection and processed for immunofluorescence staining to reveal presynaptic motor axons and terminals (magenta) along with BTX to stain postsynaptic densities (cyan). Bar = 40 μm. **b** Control and nerve injured animals were subcutaneously injected in vivo in the head/neck region with Alexa-488 BTX (cyan, BTX-1). After 7 days, LAL muscles were dissected and incubated with Alexa-555 BTX (magenta, BTX-2). **c** Following the “two-color BTX assay” NMJs were categorized as “stable” if BTX-1 and BTX-2 labels were similarly intense or “unstable” if BTX-2 intensity was comparatively higher. **d** LAL muscles from control mice and 5, 7, 8, 9, 10, and 11 days after nerve crush injury were stained for presynaptic motor axons and terminals (magenta) and with BTX to stain postsynaptic densities (cyan). Bar = 20 μm. **e**, **f** The NMJ postsynaptic domains from LAL muscles of control adult mice and 10, 14, 21, 37, 60, and 90 days after nerve crush injury were subjected to the “two color BTX assay” and NMJs at different times after nerve crush were categorized as “stable” or “unstable.” The results are represented as the mean ± SEM (N: 3–4 mice; 2 female plus 1–2 male mice). *p < 0.05; **p < 0.01; ***p < 0.001; ****p < 0.0001, Mann–Whitney test. Asterisks show significant differences between stable and unstable structures, as compared to their respective controls

We next sought to analyze the stability of AChRs within the endplate, as its rapid removal from the postsynaptic membrane is a hallmark of NMJ denervation [10]. With this aim, we followed an in vivo two-color BTX method [31] by which postsynaptic AChRs were labeled in vivo with a non-saturating dose of a fluorescently tagged BTX (BTX-1) and after dissection, the LAL muscles were labeled with a different fluorescently tagged BTX (BTX-2). Using confocal microscopy, AChR aggregates were categorized as “stable” if BTX-1 and BTX-2 labels were of similar intensity or as “unstable” if BTX-1 labeling was mainly absent and BTX-2 intensity was comparatively higher. Considering that after chronic denervation most postsynaptic domains lose their pretzel-like morphology and exhibit a fragmented morphology (Fig. 4a), and ectopic AChRs reach a significant proportion a month later (Fig. 1d), we performed these experiments in a time frame covering 15 days post nerve resection. In control muscles, as AChR removal from the postsynaptic membrane was around 50% of the total AChRs (Fig. 4b, Ctrl), most postsynaptic structures were classified as stable (Fig. 4c). As expected, the proportion of stable postsynaptic structures decreased after denervation, concomitantly with an increase of unstable structures (Fig. 4b, c). To perform similar analyses during NMJ regeneration, we first characterized the time point of NMJ reinnervation in the LAL muscle (Fig. 4d). We found that regenerated axons reached the LAL muscle by 7 days after nerve crush, while different degrees of partial NMJ reinnervation were observed in the period between 8 and 10 days after injury. At 11 days, most postsynaptic domains were fully reinnervated (Fig. 4d). Interestingly, our two-color BTX analyses showed a significantly higher proportion of unstable postsynaptic domains between 10 and 21 days after nerve injury (Fig. 4e, f). While AChR stability tended to recover 37 days after nerve crush injury, a slight but statistically significant biphasic behavior was observed after 60 days. Finally, at 90 days, the percentage of unstable and stable structures was comparable to uninjured control muscles (Fig. 4f). Our data reveal that postsynaptic stability is highly impaired upon chronic denervation of the LAL muscle. Interestingly, postsynaptic stability is not immediately recovered after NMJ reinnervation, as this parameter is rather delayed by a time period of several weeks and only recovers control values three months after damage.

### Short-term denervation leads to a long-term adaption of the NMJ structure

**Fig. 5:**
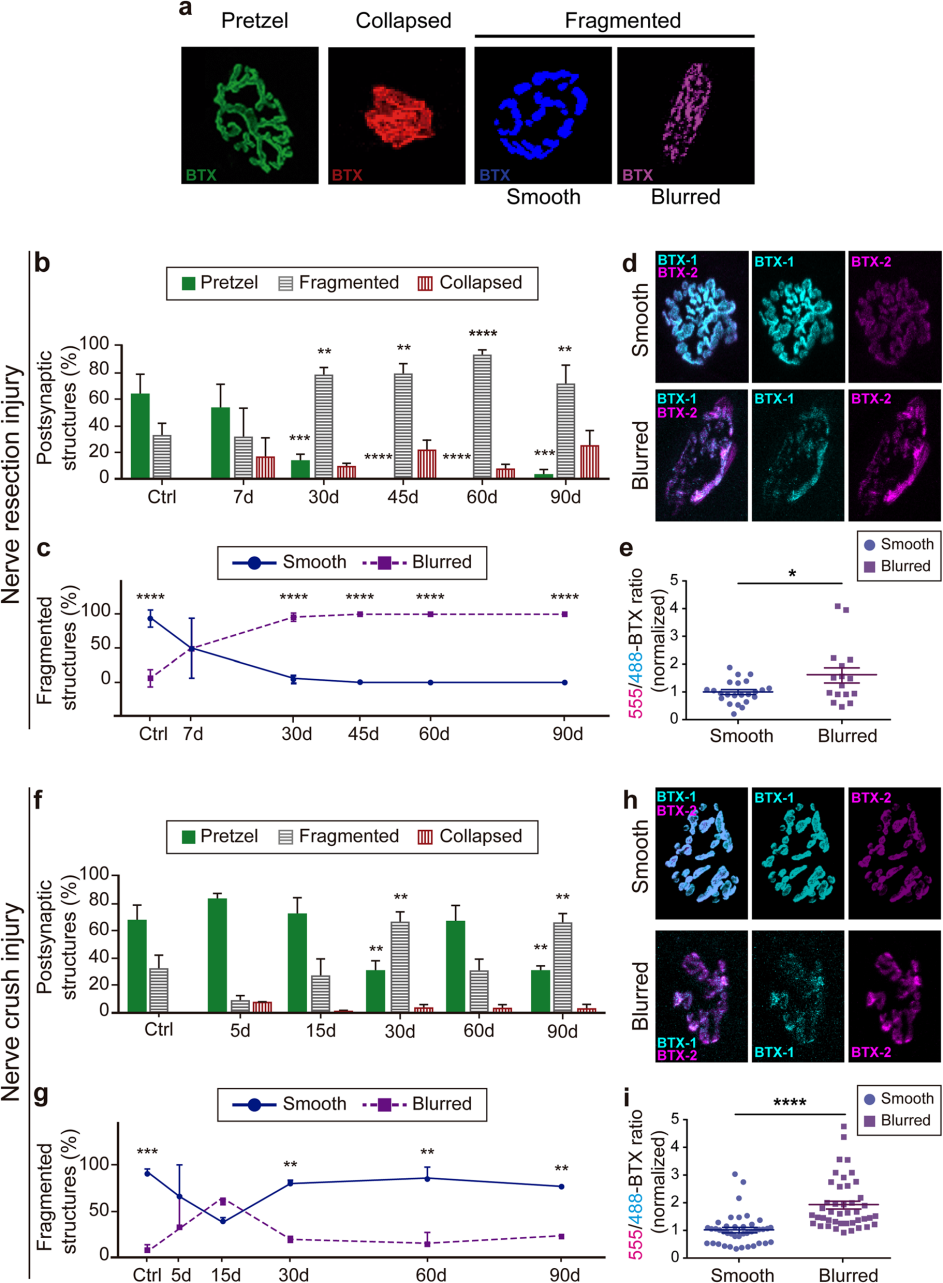
Alterations in the NMJ postsynaptic domain after nerve damage. **a** Different morphologies of the NMJ postsynaptic apparatuses were categorized as mature pretzel-like (green), collapsed (red), and fragmented morphologies that were in turn sub-classified as fragmented smooth (blue) and fragmented blurred (purple). **b** The proportion of pretzel-like (green bars), fragmented (grey bars), and collapsed (red bars) NMJ postsynaptic morphologies was calculated in control mice and 7, 30, 45, 60, and 90 days after nerve resection. **c** The proportion of smooth (blue line) and blurred fragmented (purple dashed line) morphologies in LAL muscles from control and nerve-injured mice at different times are expressed as the percentage of total fragmented NMJs. **d** The stability of postsynaptic domains of smooth (upper panel) and blurred fragmented (lower panel) structures after nerve resection was calculated after the two-color BTX assay. **e** Fluorescence intensity ratio between BTX-2 and BTX-1 of smooth and blurred fragmented morphologies after nerve resection injury. Values were normalized to the average ratio of smooth fragmented structures. **f** The proportion of pretzel-like (green bars), fragmented (grey bars), and collapsed (red bars) NMJ postsynaptic morphologies was calculated in control mice and at different times after nerve crush injury. **g** Smooth (blue line) and blurred fragmented (purple dashed line) morphologies were quantified in LAL muscles from control and nerve-injured mice at different times and are expressed as the percentage of total fragmented NMJs. **h** The stability of postsynaptic domains of smooth (upper panel) and blurred fragmented (lower panel) structures after nerve cut was calculated after the two-color BTX assay. **i** Fluorescence intensity ratio between BTX-2 and BTX-1 of smooth and blurred fragmented morphologies after nerve crush injury. Values were normalized to the average ratio of smooth fragmented structures. The results are represented as the mean ± SEM (N: 3–4 mice; 2 female plus 1–2 male mice). For the stability analysis more than 30 NMJs of 3–4 different animals were quantified in each condition. **p < 0.01; ***p < 0.001; ****p < 0.0001, two-way ANOVA (**b**, **c**, **e**, **g**), or t-test (**d**, **i**)

As the organization of AChR aggregates within the endplate is altered in conditions affecting NMJ integrity and function, we next sought to analyze postsynaptic morphology after nerve damage. AChR clusters were categorized into mature pretzels (complex and highly branched shapes), collapsed (shrunk, non-branched structures), and fragmented pretzels (i.e., those having more than six distinctive AChRs aggregates or “fragments”) (Fig. 5a). Soon after facial nerve resection, collapsed structures (Fig. 5b; red bars) begin to appear, concomitant to a marked decrease of pretzel-like shapes (Fig. 5b; green bars), resulting in their absence from 45 days after nerve resection onwards. After 30 days, most postsynaptic domains become fragmented (Fig. 5b; gray bars). A detailed observation allowed us to identify and quantify two different organization patterns of fragmented postsynaptic structures that, according to previous evidence [32], were classified as (i) fragmented smooth, exhibiting discrete fragments with defined edges, and (ii) fragmented blurred, as those having multiple small fragments displaying diffuse edges (Fig. 5a). In chronically denervated NMJs, we found an evident prevalence of fragmented blurred morphologies from as early as 7 days (around 50%) to 30 days onwards (> 90%) (Fig. 5c). To complement the idea that different forms of NMJ fragmentation arise from NMJ degeneration, we analyzed AChR stability in both types of fragmented structures (Fig. 5d). We found that fragmented blurred structures are unstable, evidenced by a strong reduction of BTX-1 detection compensated by higher BTX-2 staining (Fig. 5e).

In the NMJ reinnervation paradigm, mature pretzels displayed a marked biphasic behavior, as their proportion decrease at 30 and 90 days after nerve crush injury, with partial recovery at 60 days (Fig. 5f; green bars). These changes mirrored an inverted biphasic behavior of fragmented structures (gray bars) (Fig. 5f). Remarkably, after a discrete period showing a similar proportion of both fragmented structures (15 days after nerve crush), we found that opposite to chronic denervation, the proportion of fragmented smooth structures become significantly higher upon NMJ reinnervation (Fig. 5g). As in the chronic denervation paradigm, fragmented blurred structures displayed higher instability than smooth fragmented ones (Fig. 5h, i). Together, these studies reveal the existence of different types of NMJ postsynaptic fragmentation, whose relative abundance likely correlates with their reinnervation potential.

**Fig. 6:**
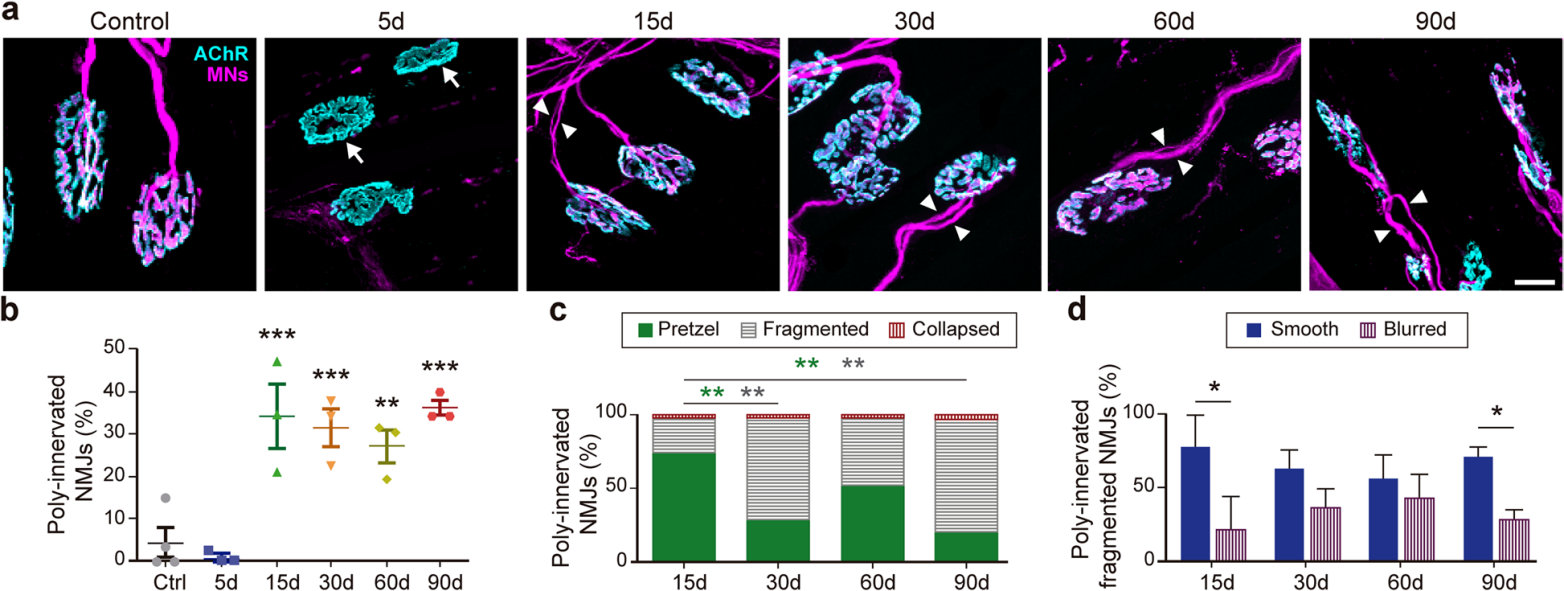
Long-lasting poly-innervation after NMJ short-term reinnervation of the LAL muscle. LAL muscles from control adult mice and from different times after nerve crush injury were dissected and subjected to immunofluorescence staining to reveal presynaptic motor axons and terminals (magenta) along with Alexa 488-BTX (cyan) to stain postsynaptic densities. **a** Representative images of denervated postsynaptic domains (arrows) at 5d and multiple axons innervating one postsynaptic domain after reinnervation (arrowheads) at different times after NMJ reinnervation. Bar = 20 μm. **b** The proportion of poly-innervated NMJs in LAL muscles from control and nerve-injured mice at the indicated times was quantified and expressed as the percentage of total NMJs. **c** Poly-innervated NMJs exhibiting pretzel-like (green), fragmented (gray), and collapsed (red) morphologies from control and nerve-injured mice at the indicated times were quantified and expressed as the percentage of total poly-innervated NMJs per animal. Green and gray asterisks show significant differences between pretzel-like and fragmented morphologies, respectively. **d** Poly-innervated NMJs displaying smooth (blue bars) and blurred fragmented (purple bars) morphologies in LAL muscles from control and nerve-injured mice at different times are expressed as the percentage of total fragmented NMJs. The results are represented as the mean ± SEM (**c**, **d**) along with each individual value (**b**) (N: 3–4 mice; 2 female plus 1–2 male mice). *p < 0.05, **p < 0.01, ***p < 0.001, one-way ANOVA test (**b**), or two-way ANOVA (**c**, **d**)

Previous findings in hind-limb muscles showed transient NMJ poly-innervation during reinnervation [5]. To evaluate whether NMJ regeneration at the LAL muscle also exhibited this parameter, we used 3D projections of the z-stacks obtained from confocal microscopy to quantify mono- and poly-innervated NMJs, as 2D images often do not suffice to distinguish between these two types of innervation (an additional figure shows this in more detail (see Additional file 1) as well as additional movie files (see Additional files 2, 3, 4 and 5)). According to previous reports, we observed that early NMJ reinnervation was accompanied by poly-innervation, as 34.30 ± 13.01% (***p < 0.001, one-way ANOVA test) of NMJs were innervated by more than one motor axon at day 15 (Fig. 6a). Remarkably, a similar proportion of NMJ poly-innervation persists until 3 months after facial nerve crush (Fig. 6b). When we analyzed NMJ poly-innervation in the different postsynaptic morphologies, we found that the initial abundance of poly-innervated postsynaptic pretzel-like shapes (Fig. 6c; green bars) turned into an increased proportion of poly-innervated fragmented structures 30 and 90 days after nerve injury (Fig. 6c; gray bars). From these, poly-innervation was significantly higher in fragmented NMJs exhibiting smooth morphology 15 and 90 days after nerve injury (Fig. 6d; blue bars).

Altogether, our results indicate that some morphometric parameters of the NMJ, including AChR aggregates area, endplate diameter, and postsynaptic stability, are altered soon after denervation and remain altered long after NMJ reinnervation. Moreover, other morphometric parameters, including the fragmentation of postsynaptic structures and marked poly-innervation seem to remain irreversibly altered, suggesting that nerve injury leads to an adaptive reminiscence of the NMJ after damage.

### Delayed functional recovery of the neuromuscular synaptic transmission after endplate reinnervation

**Fig. 7:**
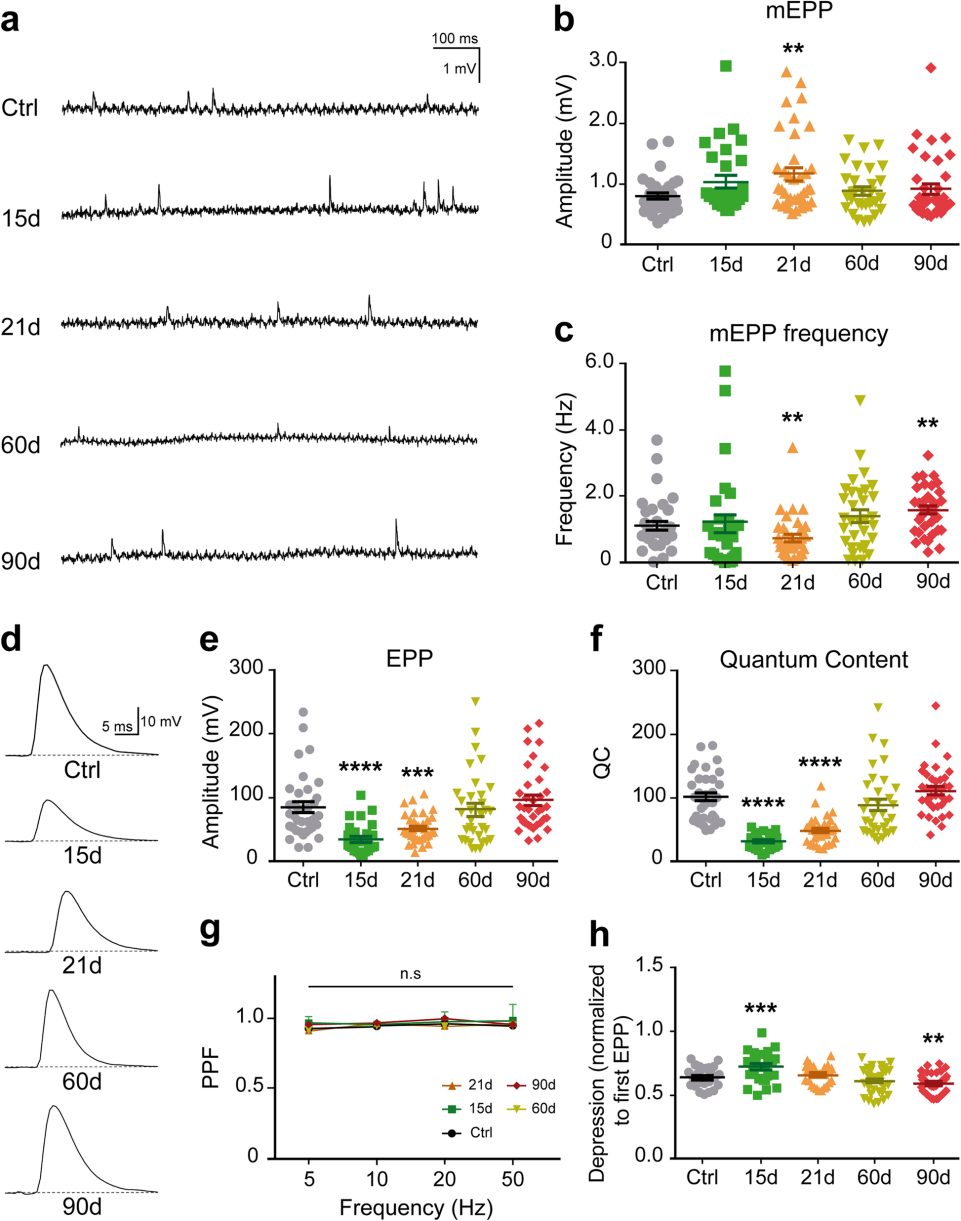
Functional analyses of the LAL muscle NMJ after nerve crush injury. For functional studies, LAL muscles were analyzed through electrophysiological intracellular recording at the indicated times after nerve crush injury. LAL muscles from age-matched mice were used as controls. **a**–**c** After blocking muscle contraction, representative mEPP traces after 0.5 Hz stimuli of NMJs from control and reinnervated muscle fibers and quantification of the amplitude (**b**) and frequency (**c**) of mEPPs from muscles from control mice and mice 15, 21, 60, and 90 days after nerve crush. **d** Representative EPP traces after 0.5 Hz stimuli of NMJs from control and reinnervated muscle fibers. **e**, **f** Quantification of EPP amplitude (**e**) and quantal content (**f**) at the NMJs of control and nerve-injured mice at the indicated times after nerve crush injury. **g** Stimulation trains of 5, 10, 20, and 50 Hz showed no changes in paired-pulse facilitation (PPF). **h** The depression index was calculated after 80 stimuli at 50 Hz. N: number of mice (N: 2 male plus 1 female mice); n: number of fibers (27-37 fibers per mouse). The results were represented as the mean ± SEM, and each individual value for each condition. **p < 0.01; ***p < 0.001; ****p < 0.0001, Mann–Whitney test (**b**, **c**, **e**, **f**, **h**), or two-way ANOVA (**g**)

To study how the sustained effects observed on both NMJ morphology and stability after denervation impact on NMJ functionality, we studied synaptic transmission at different time points after nerve crush injury in ex vivo nerve–muscle LAL preparations using intracellular electrophysiological recordings. We analyzed spontaneous miniature endplate potentials (mEPPs) and evoked endplate potentials (EPPs); we also obtained information on quantal content (QC, the number of quanta released per action potential) and possible changes in short-term plasticity (facilitation and depression) during repetitive stimuli. The average amplitude of mEPPs was increased 21 days after injury (Fig. 7a, b), while their frequency decreased at 21 days and increased at 90 days (Fig. 7a, c). The mean amplitude of EPPs was significantly reduced 15 days (34.64 ± 4.37 mV; ****p < 0.0001, Mann–Whitney test) and 21 days (51.23 ± 3.53 mV; ***p < 0.001, Mann–Whitney test) after nerve crush injury, compared to uninjured controls (85.21 ± 8.12 mV) (Fig. 7d, e). This evoked response recovered control values 2 months after injury (Fig. 7d, e), consistent with our observations that mice begin to recover ear movement one week after nerve crush. Similarly, the QC decreased 15 and 21 days after nerve damage but recovered 60 days after nerve injury (Fig. 7f).

No differences in paired-pulse facilitation (PPF) at frequencies ranging from 5 to 50 Hz were observed between control and muscles 15, 21, 60, and 90 days after denervation (Fig. 7g), indicating no differences in the probabilities of neurotransmitter release. In contrast, short-term depression of EPP amplitude was slightly but significantly increased shortly after NMJ reinnervation (Fig. 7h), suggesting a subjacent decline of the QC under continuous stimuli which, among other possibilities, could be explained by a decrease in the refilling of the readily releasable pool (RRP) of vesicles. This parameter was also slightly decreased 3 months after nerve injury (Fig. 7h).

**Fig. 8:**
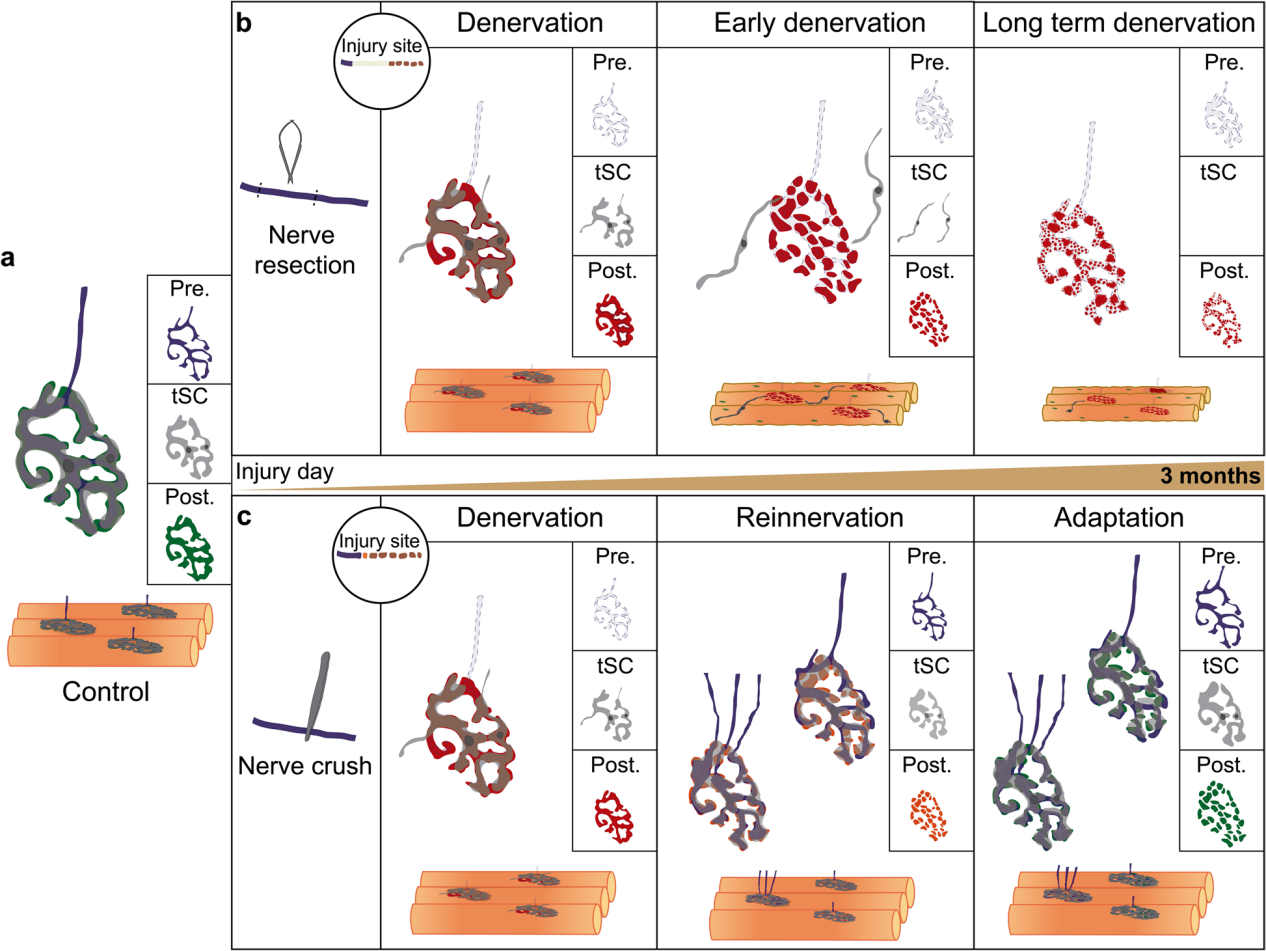
Morphological adaptation of the NMJ after degenerative and regenerative damage. **a** In the NMJ of control muscles, the postsynaptic domain (green) exhibits a complex pretzel-like morphology, mostly covered by a single presynaptic axon terminal (blue), and 2 or 3 terminal Schwann cells (gray) covering the endplate. **b** After nerve resection (NMJ degeneration), the distal axon stump goes through Wallerian degeneration (circle), tSC extend projections outside of the synaptic region (middle panel), the postsynaptic domain become denervated, unstable, and fragmented (red), ectopic AChR aggregates appear along the muscle fiber, and muscle fibers suffer atrophy (middle and right panel). **c** After nerve crush injury (NMJ regeneration), the distal axon stump goes through Wallerian degeneration (circle) and the postsynaptic domain becomes denervated and unstable (left panel). Reinnervation results in a significant mono- and poly-innervated synapses (middle panel), while most postsynaptic domains become fragmented and do not recover the control values of stability (orange postsynaptic domain in the middle panel). Long after reinnervation, a significant proportion of NMJs are still poly-innervated (~ 30%). While postsynaptic domains recover their stability, most of them remain fragmented (right panel)

In summary, our findings reveal that although degenerative and regenerative paradigms of nerve injury led to morphological and functional alterations of the NMJ, some of them are efficiently recovered after reinnervation (Fig. 8). Other parameters, including AChR aggregates area, nerve terminal area and perimeter, endplate diameter, and postsynaptic stability, were recovered only three months after nerve injury. Importantly, NMJ poly-innervation and fragmentation remained altered long after muscle reinnervation has been accomplished (Fig. 8). Despite the observed long-term consequences of short-term reinnervation, synaptic transmission at the NMJ is recovered to control levels 2 months after nerve crush injury, suggesting that the observed altered morphological features are part of adaptive mechanisms that take place during the regenerative process.

## Discussion

Although the peripheral nervous system bears higher regenerative capabilities than central synapses [3], motor function recovery is often ineffective in humans [33]. Possible explanations are the longer distances that regenerating human motor axons must travel in comparison to rodents ones, along with the similar (or even slower) axonal growth rate observed in humans compared to rodents [33]. Moreover, although significant advances have been made in microsurgical techniques used for facial nerve repair, the recovery of motor function has shown results varying from excellent to sub-optimal, being fair-to-good results higher in proportion [9, 24]. Interestingly, studies using different paradigms of peripheral nerve damage have shown that morphological regeneration of the NMJ does not necessarily correlate with motor function recovery [22]. Likely, the functional rescue of neuromuscular synapses observed in murine species is limited by the denervation time frame and depends on the ability of the cellular components of the neuromuscular synapse to establish a permissive niche for NMJ regeneration [2]. In this context, our present findings show that NMJ reinnervation of the LAL muscle is accompanied by long-lasting changes in its three cellular components.

Interestingly, some of the long-lasting changes observed after short-term reinnervation are also evident in the NMJs of other physiological and pathological conditions. For instance, several studies reveal that nerve damage-induced NMJ regeneration mimics some of the molecular and cellular events of embryonic NMJ development. In the postsynaptic domain, early NMJ formation is characterized by a pre-pattern of AChR plaques in still denervated muscle fibers, some of which will become innervated and mature into pretzel-like structures [34]. As previously shown [27], denervated LAL muscle fibers exhibited ectopic AChR plaques that disappear once reinnervation is achieved, in line with evidence showing that neuronal motor activity and the subsequent increase of muscle intracellular calcium inhibit the expression of the AChR subunits, allowing the establishment and localization of AChR aggregates specifically in the synaptic region of the muscle fiber [35]. We also observed a decrease in the area of AChR aggregates after nerve damage, a phenotype that has been attributed to muscle fiber atrophy [4]. However, our findings show that while NMJ reinnervation occurred 11 days after injury, it correlated with decreased AChR aggregates area even two months after injury. Similarly, we also found that early instability of surface AChRs [10] persisted long after incoming motor axons established synaptic contacts with denervated muscle postsynaptic domains.

An archetypical hallmark of NMJ alterations is postsynaptic fragmentation. Indeed, endplate denervation triggers a sequence of events resulting in decreased AChR half-life in the postsynaptic membrane, reduced postsynaptic density, and the subsequent loss of postsynaptic regions [36]. Interestingly, NMJ fragmentation has been described not only after nerve injury-dependent denervation, but also in the NMJs of muscle and motor disease models [37, 38], and it is considered a distinctive feature of aged NMJs [17]. Together with previous findings showing some heterogeneity in the classification of fragmented NMJs [32], the occurrence of NMJ fragmentation in diverse conditions suggests that this parameter accounts for a variety of phenotypes. Interestingly, our analyses on NMJ morphology and stability allowed discriminating a stable smooth fragmented morphology, observed mainly upon functional recovery, and an unstable blurred fragmented morphology, predominant in NMJs denervated by facial nerve resection, suggesting that these structures are related to the capability of the postsynaptic domain to recover functionality upon reinnervation. In this regard, smooth NMJ fragmentation caused by nerve damage could be part of an adaptive mechanism comparable to that occurring during aging, both aimed to maintain motor function. It has been argued that NMJ fragmentation could appear in aged murine species due to their rapidly prolonged life span and the subsequent lack of age-specific adaptive cellular mechanisms to preserve motor function [39]. Conversely, human NMJs, which are significantly more fragmented and smaller than mouse NMJs, showed almost no morphological changes along their life span, remaining stable regardless of the aging process [20]. In addition, a recent clinical report described a patient that exhibited denervated muscles 5 months after nerve injury, with NMJs having normal gross shape but smaller size than controls, that were functionally recovered  one year after nerve transfer therapy [8]. Although the likelihood to obtain successful results after nerve damage is higher in rodents than in humans [3], this evidence correlates the higher resilient capacity of the human NMJ with its natural fragmentation, as murine pretzel-like mature neuromuscular synapses fail to recover motor function after a critical time period of denervation [22]. As our present results show that the proportion of unstable structures decreases only when the abundance of fragmented postsynaptic structures increases, one interesting possibility is that stable smooth postsynaptic fragments represent a morphological adaptation that somehow improves the chances of functional NMJ reinnervation.

Although reinnervation occurs relatively fast in our model, the proportion of poly-innervated NMJs remained significantly increased for up to three months after nerve damage. This observation is consistent with experiments showing that some facial muscles maintain a significant percentage of poly-innervated NMJs (~ 16%) after muscle reinnervation [40]. Moreover, in some frog species, there is a significant percentage of poly-innervated muscle fibers in adult stages [41]. Also, during embryonic NMJ development, immature plaque-like AChR aggregates are mainly poly-innervated in contrast to mature pretzel-like mono-innervated aggregates [18]. In our experiments, we found that early after reinnervation, most poly-innervated NMJs have a pretzel-like shape whereas after 3 months poly-innervated NMJs are mostly fragmented, suggesting that poly-innervation leads to postsynaptic fragmentation. Of note, we assessed NMJ poly-innervation based on previous confocal microscopy-based imaging after immunohistochemistry staining [19, 42], while clearer and more definite conclusions have been drawn from elegant experiments performed in transgenic animals having motor neurons that express different fluorescent proteins [43]. Although we improved our analyses using 3D-reconstructed images to distinguish NMJs having multiple innervations, our approach may not efficiently discriminate axonal sprouting from true poly-innervation. Despite these experimental limitations, the idea that increased poly-innervation or sprouting is related to NMJ fragmentation as an adaptive functional response after reinnervation is supported by our functional experiments showing that reinnervated muscle fibers exhibit normal synaptic transmission since two months after nerve injury. The hypothesis that NMJ fragmentation is associated with a decline in the efficacy of neuromuscular transmission has been thoroughly challenged by comparing muscles from middle-aged (2.6% of highly fragmented NMJs) and aged (22% of highly fragmented NMJs) animals [44]. By correlating key functional parameters with the number of fragments in individual fibers, the authors showed that highly fragmented NMJs from old mice had similar or even higher endplate current (EPC) amplitude than those from middle-aged mice [44]. Despite not being performed in isolated muscle fibers, our experiments showed that synaptic transmission in LAL muscles containing almost 80% of fragmented and more than 30% of multiple innervated NMJs exhibited similar synaptic transmission values than control non-injured muscles. Together, these findings show that two conditions resulting in increased NMJ fragmentation—aging and NMJ reinnervation after nerve injury—correlate with normal synaptic transmission, reinforcing the notion that rather than an indicator of NMJ functional decline, this morphological alteration likely represents an adaptive response for functional recovery. Interestingly, a comparative study has shown that the NMJs of the LAL muscle are particularly resistant to aging and pathologies [45]. In this regard, it is still an open question if the NMJs of more active or critical muscles for the mobility of organisms adapt differently to denervation and reinnervation.

Regarding tSCs, we detected and quantified tSC projections in both nerve damage models and we did not find significant differences in the sprouting of tSCs upon NMJ reinnervation comparable to those observed in hind-limb muscles after sciatic nerve crush injury [7]. These findings could be related to the proximity of the facial nerve damage to the LAL muscle in our model and its subsequent faster reinnervation; however, previous studies have shown that nearly 20% of the NMJs have tSCs bearing projections [15]. We also found that chronic NMJ denervation resulted in fewer tSCs at the NMJ and SCs outside the NMJ, suggesting that denervation-induced tSC sprouting and migration outside the synaptic region give rise to tSC death when axonal inputs are not present. However, previous studies have shown isolated SCs in atrophied bands of Büngner in distal nerve stumps as well as SCs in intramuscular distal nerve stumps even more than 2 years after nerve injury [46], revealing that SCs have the potential to survive without axonal inputs for long periods of time.

## Conclusions

Altogether, our results reveal long-term effects on NMJ morphology, stability, and function after short-term denervation and subsequent reinnervation. This suggests that after nerve damage, a reminiscence of the NMJ is induced, which can be proposed as a rearrangement of the cellular components of this synapse upon nerve injury in order to allow for proper functional recovery. The ability of the cellular components of the neuromuscular synapse to establish a permissive niche for NMJ regeneration could be manifested in morphological adaptations that account for the capacity of the denervated muscle to recover motor function upon reinnervation. These new findings could be relevant to develop tools to diagnose whether denervated NMJs in humans would be able to recover motor function and to eventually manipulate the behavior of the different NMJ cellular parties to accomplish successful muscle reinnervation.

## Methods

### Animals

All CF-1 and FVB mice in this study were maintained at 20–26 °C, with dark/light cycles of 12 h, and fed with pellet (Prolab RMH-3000, LabDiet) and water ad libitum. Mice were sacrificed by an overdose of inhalatory isoflurane anesthesia. Experimental procedures were approved by the Bioethics Committee at Universidad de Concepcion, Chile and followed the norms imposed by the Bioethics Committee of the National Research and Development Agency, Chile (ANID), as well as the guidelines of the European Council Directive for the Care of Laboratory Animals.

### Facial nerve injury

Two facial nerve injury protocols were performed [23] leading to either chronic denervation or NMJ reinnervation. Briefly, male and female adult mice (3–6 months old) were anesthetized by isoflurane inhalation (2.5% v/v isoflurane with a 0.8-1 L/min oxygen mixture). After shaving the right ear posterior region, a surgical 5-mm skin incision was performed to expose the facial nerve branches. The posterior auricular branch of the facial nerve, which innervates the LAL muscle, was carefully cleared avoiding direct manipulation. For the chronic denervation protocol, a 5-mm portion of the facial nerve branch was transected. For the reinnervation protocol, the facial nerve branch was crushed during 30 s using Dumont #5/45 forceps (Fine Science Tools). Finally, the skin was sutured using absorbable monofilament surgical suture (Ethicon Vicryl USP 6-0), and animals were monitored until their recovery. Control experiments only considered skin incision and facial nerve exposure.

### NMJ staining, imaging, and analyses

Whole-mounted LAL muscles were fixed as described [23, 31]. After washing with 0.01M PBS/0.5% v/v Triton X-100 for 2 h, samples were incubated with 0.1M glycine in PBS for 30 min. Blocking was performed with 4% BSA dissolved in PBS/0.5% Triton X-100. Primary antibodies against neurofilaments (2H3, 1:300), synaptic vesicles (SV2, 1:200) (Cat # AB_2314897 and AB_2315387; both from the Developmental Studies Hybridoma Bank, DSHB, University of Iowa Department of Biology, IA, USA), and S100B (1:300) (Cat # Z0311; DAKO, Santa Clara, CA, USA) were incubated overnight in blocking solution (PBS/0.5% Triton X-100/4% BSA). After washing, samples were incubated with the secondary antibodies (Cy3 1:250; Cy5 1:250; Donkey H+L, Jackson Immunoresearch Laboratories, West Grove, PA, USA) along with Alexa488-conjugated α-bungarotoxin (BTX, Molecular Probes; 1:500) overnight at 4 °C and subsequently mounted between two coverslips in DAKO fluorescence medium. Images were acquired using a LSM 700 laser scanning confocal microscope (CMA BioBio, Universidad de Concepción). Confocal z-plane optical sections (1 μm intervals) were captured with a Plan-Apochromat 40X/1.3 Oil DIC M27 objective. To adjust the fluorescence intensity in the deepest z-planes without varying the power of the laser scanning, the “auto z brightness correction” was used, as it allows an automatic and linear interpolation of values among neighboring positions within the z stack. The NMJ morphometric analyses were performed as described [23, 31, 47]. Ectopic AChR aggregates were identified as plaque or oval morphologies and by their distribution within muscle fibers, as we have previously described [48]. To quantify poly-innervation as well as tSC number and projections, z-stacks were projected in 3D using the ImageJ software. Endplates were considered poly-innervated when at least two sources of innervation, either axons or thin sprouts, contact them, as described [19, 42]. Postsynaptic domains morphology (pretzel, fragmented and collapsed, as well as fragmented smooth and blurred) was classified manually by an experienced investigator blind to the experimental groups.

### Two-color bungarotoxin assay

To analyze AChRs dynamics, isoflurane-anesthetized mice were subjected to subcutaneous injection (in the head/neck region) of a non-saturating concentration (4 μg/mL in sterile 0.01 M PBS) of Alexa-488 conjugated BTX (BTX-1). Seven days later, mice were sacrificed and the LAL muscles were dissected, pinned to a Sylgard coated dish, and fixed with 0.5% v/v formaldehyde (Merck-Millipore) for 90 min at room temperature. After fixation, the muscles were washed, and newly incorporated AChRs at muscle surface were labeled with Alexa-555 conjugated BTX for 60 min (BTX-2, 2 μg/mL in 0.01 M PBS). Images (z-stacks) were collected at 1 μm intervals in a Zeiss LSM 700 confocal microscope (CMA BioBio, Universidad de Concepción). All acquisition parameters were equally maintained between experiments. Pretzel-like structures were manually traced using the Image J software and the mean fluorescence intensity of BTX-1 and BTX-2 was quantified. In our working conditions, NMJs were classified as “unstable” when the BTX-2/BTX-1 ratio was ≥1.0 or as “stable” for values < 1.0, as we have previously described [48]. Data are expressed as the percentage of stable and unstable AChRs at denervated NMJs in both nerve injury protocols.

### Electrophysiological intracellular recording

Ex vivo preparations of LAL muscles containing an intact 5 mm facial nerve stump were transferred to the stage of an Olympus BX50WI upright microscope and continuously perfused with an external solution (in mM: 135 NaCl, 5 KCl, 1 MgCl_2_, 12 NaHCO_3_, 12 glucose, and 2 CaCl_2_) at room temperature. Evoked (EPP) and spontaneous miniature (mEPP) endplate potentials were recorded and analyzed as described [49]. Briefly, the nerve was stimulated through 0.2 ms square-wave pulses at the indicated frequencies using a suction electrode. A glass micropipette filled with 3 M KCl was connected to an intracellular recording amplifier (Neuro Data IR283, Cygnus technology, Southport, NC, USA) through a chloride silver wire and used to impale single muscle fibers near the motor nerve endings. Muscle contraction was prevented by including in the bath 3–4 μM μ-conotoxin GIIIB (Alomone Laboratories, Jerusalem, Israel), a specific blocker of muscular voltage-gated sodium channels. Data were analyzed as previously described [49]. EPP amplitudes were normalized to − 70 mV and corrected for nonlinear summation.

### Statistical analyses

All data are presented as mean ± SEM and analyzed using GraphPad Prism 6. Differences among control and denervated muscles were assessed using Mann–Whitney test, *t*-test, and one-way or two-way ANOVA. A *p* value of < 0.05 indicates statistically significant differences. Figures were made using Adobe Illustrator CS6. Datasets with normal distribution were analyzed with parametric statistical tests (*t*-test or ANOVA), while datasets not having normal distribution were analyzed with the Mann–Whitney non-parametric test. The variance was similar between datasets. All the individual data values for each graph are provided in Additional file 6.

## Supplementary Information

**Additional file 1: Fig. S1.** NMJ innervation profiles. The upper panels show a single innervated (left) and a poly innervated NMJ (right). Lower panels show NMJs that seem innervated by two motor axons (left) and an NMJ that seems innervated by a single motor axon (right) analyzed through maximum intensity projection of the z-stacks.

**Additional file 2: Movie 1.** A single innervated NMJ observed through 3D projection.

**Additional file 3: Movie 2.** A poly-innervated NMJ observed through 3D projection.

**Additional file 4: Movie 3.** The NMJ that seems innervated by two motor axons (solid arrowheads) from Additional file 1 (lower left panel) is shown in a 3D projection. The rotation shows that only one motor axon innervates the postsynaptic domain while the second one does not (empty arrowheads).

**Additional file 5: Movie 4.** The NMJ that seems innervated by a single motor axon (empty arrowhead) from Additional file 1 (lower right panel) is shown in a 3D projection. The rotation shows that two motor axons innervate the postsynaptic domain (solid arrowheads).

**Additional file 6.** Individual data values.

## Data Availability

All data generated or analyzed during this study are included in this published article and its supplementary information files. The raw microscopy datasets are available from the corresponding author on reasonable request.

## Abbreviations

NMJ: Neuromuscular junction
AChR: Acetylcholine receptor
tSC: Terminal Schwann cells
LAL: Levator auris longus
BTX: Bungarotoxin
EPP: Evoked endplate potential
mEPP: Miniature endplate potential
