# Supplementary figures and images for: Functional regeneration of the murine neuromuscular synapse relies on long-lasting morphological adaptations
Source: BMC Biol. 2022 Jul 8;20:158. doi: 10.1186/s12915-022-01358-4 (PMC9270767; doi:10.1186/s12915-022-01358-4)

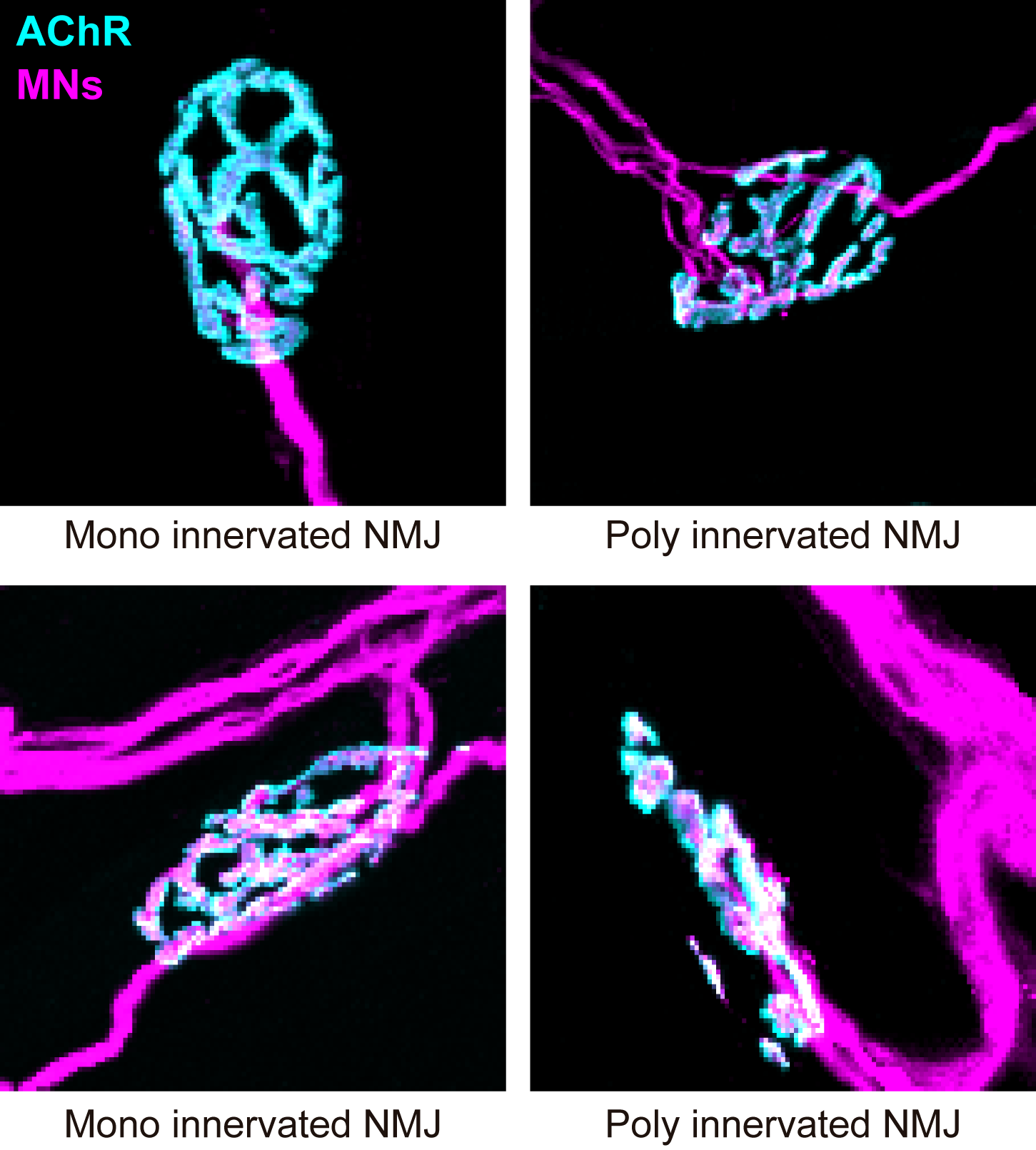

Supplement: Supplementary file 1 — Additional file 1: Fig. S1. NMJ innervation profiles. The upper panels show a single innervated (left) and a poly innervated NMJ (right). Lower panels show NMJs that seem innervated by two motor axons (left) and an NMJ that seems innervated by a single motor axon (right) analyzed through maximum intensity projection of the z-stacks. [file 12915_2022_1358_MOESM1_ESM.png]
